# The Stem Cell Population of the Human Colon Crypt: Analysis via Methylation Patterns

**DOI:** 10.1371/journal.pcbi.0030028

**Published:** 2007-03-02

**Authors:** Pierre Nicolas, Kyoung-Mee Kim, Darryl Shibata, Simon Tavaré

**Affiliations:** 1 Unité Mathématique Informatique et Génome UR1077, Institut National de la Recherche Agronomique, Jouy-en-Josas, France; 2 Department of Pathology, Keck School of Medicine, University of Southern California, Los Angeles, California, United States of America; 3 Program in Molecular and Computational Biology, Department of Biological Sciences, University of Southern California, Los Angeles, California, United States of America; 4 Department of Oncology, University of Cambridge, Cambridge, United Kingdom; Fred Hutchinson Cancer Research Center, United States of America

## Abstract

The analysis of methylation patterns is a promising approach to investigate the genealogy of cell populations in an organism. In a stem cell–niche scenario, sampled methylation patterns are the stochastic outcome of a complex interplay between niche structural features such as the number of stem cells within a niche and the niche succession time, the methylation/demethylation process, and the randomness due to sampling. As a consequence, methylation pattern studies can reveal niche characteristics but also require appropriate statistical methods. The analysis of methylation patterns sampled from colon crypts is a prototype of such a study. Previous analyses were based on forward simulation of the cell content of the whole crypt and subsequent comparisons between simulated and experimental data using a few statistics as a proxy to summarize the data. In this paper we develop a more powerful method to analyze these data based on coalescent modelling and Bayesian inference. Results support a scenario where the colon crypt is maintained by a high number of stem cells; the posterior indicates a number greater than eight and the posterior mode is between 15 and 20. The results also provide further evidence for synergistic effects in the methylation/demethylation process that could for the first time be quantitatively assessed through their long-term consequences such as the coexistence of hypermethylated and hypomethylated patterns in the same colon crypt.

## Author Summary

The dynamics of the stem cell populations in human colon crypts are of interest to cancer researchers and stem cell biologists alike. One approach to studying stem cell divisions would be to adopt methods from population genetics: cells are sampled from crypts, DNA markers such as single nucleotide polymorphisms are identified, and a model of how these mutations arose is used to infer aspects of the ancestry of the sample. Because cells within an individual are being studied, mutations of this sort are extremely rare, and an alternative marker has to be used. Methylation patterns provide a feasible alternative, containing information similar to that obtained from short DNA sequences. The present study shows how such data can be used to infer aspects of stem cell dynamics, including inference about the likely number of stem cells in a crypt. In addition, biological aspects of methylation and demethylation are also studied.

## Introduction

Most tissues are renewed during the life of the organism through a continuous replacement of their differentiated cells by new mature cells that originate from tissue-specific stem cell lineages. Genetic and epigenetic somatic variations having long-term consequences are believed to preferentially affect these cell lineages. Besides its intrinsic interest, understanding the structure of the stem cell populations is therefore expected to help gene therapy, cancer therapy, and aging research. Our knowledge about the adult tissue-specific stem cells is still sparse, but the experimental results accumulated during the past decades on several tissues from a few organisms suggest the existence of stem cell niches [1,2]. Each niche contains a small self-renewing population of proliferating cells, the stem cells, whose progeny commit to differentiation processes that span several rounds of cell division. In some tissues, such as *Drosophila* ovary and testis, cells that enter differentiation processes have been shown to be the result of asymmetric stem cell divisions giving rise to one stem cell and one cell committed to differentiation [3]. In this context, rare symmetric stem cell division events producing either two cells committed to differentiation or two stem cells may compensate for occasional gain or loss of one stem cell [4].

Cell turnover is particularly fast in the gastrointestinal epithelium where the mature differentiated cells live only a few days. In this epithelium, the self-renewing unit is a small and morphologically well-identified structure known as the intestinal crypt. Probably owing to this relatively simple anatomical structure and its rapid cell turnover, the intestinal epithelium is one of the tissues where the stem cells have been the most studied in mammals. Results have been recently reviewed in [5–7].

Each crypt contains about 2,000 cells in Human and exhibits a strong polarity with mature epithelial cells located at the extremity of the crypt opening onto the gastrointestinal tract. Phenotype markers allowing an accurate identification of intestinal stem cells are still lacking, although some progress is being made [8]. Staining experiments of cell lineages in mouse using tritiated thymidine show that cells migrate away from the base of the crypt toward the lumen while they differentiate [9,10]. The stem cell niche is therefore believed to be located at the base of the colon crypt. In the small intestine, the situation is slightly different as Paneth cells occupy the very base of the crypt (a type of mature differentiated cell absent in the colon crypt) and the stem cell niche is believed to be located just above the Paneth cells.

Gastrointestinal stem cells have a short cell cycle: they seem to divide daily in the mouse small intestine and may divide about weekly in the human colon [5,6,11]. Staining experiments with tritiated thymidine in mouse reveal asymmetric segregation of the DNA between daughter cells during stem cell division: stained template strands are retained through rounds of cell divisions at the likely location of the stem cell niche [12]. This suggests that mechanisms reducing the probability of mutations in the stem cell lineages may exist. It also provides strong support for a central role of asymmetric stem cell division in the stability of the intestinal stem cell populations. Experiments with chimeric mice and with mutagen agents have shown that crypts initially polyclonal for one marker eventually become monoclonal, which suggests that symmetric division occasionally occurs and leads to a niche succession by stochastic extinction of stem cell lineages. The time needed for a single stem cell lineage to replace all other lineages, or “clonal stabilization time,” was measured as approximately 24 weeks in the mouse small intestine but only four weeks in the mouse colon. Studies are much harder in human where mutagenesis experiments cannot be undertaken. Making use of radiotherapy-induced mutations, one study suggested that a significant fraction of the somatic mutations in human colon stem cells are lost within one year [13].

Most of the insights we have about the number of stem cells in intestinal crypts come from mouse studies where the number of crypts surviving increasing doses of radiation is measured. The underlying hypothesis of these studies is that the number of stem cells can be estimated assuming a simple model for dose-dependent cell death if crypts regenerate, as long as one stem cell survives the treatment. However, the estimated number of stem cells increase from about six to 36 as higher doses of radiation are used. It may be that a cell that would differentiate in a normal context can be recruited to regenerate the crypt [5], but alternative explanations cannot be ruled out [14]. This also emphasizes the difficulty of estimating the actual number of stem cells in normal physiological conditions by this approach. Another limitation of this approach is that it cannot be used in humans.

We recently proposed to investigate the properties of the human intestinal stem cell populations through the analysis of methylation pattern polymorphism that occurs naturally within the crypts in some CpG islands [15,16]. Methylation at CpG sites serves as cell lineage markers in these analyses as inheritable methylation changes occur somatically [17]. Compared with genetic DNA sequences that are widely used in population studies at the organism level, epigenetic methylation patterns have the advantage of evolving much faster. Even a small number of CpG sites can carry information about the underlying genealogy of cells sampled from closely related lineages. Several aspects of the approach make it particularly attractive: it does not require any treatment that could disturb the normal self-renewal dynamic of the crypt; it provides accurate discrete data richer than binary phenotype polymorphism; and it is practicable in humans.

Our previous analyses of the methylation pattern polymorphisms were based on forward simulations of the whole crypt content under a simple stem cell–niche model [15,16]. Although it provided support for a stem cell–niche model with multiple stem cell lineages whose genealogy shows coalescent events leading to stochastic niche succession during life, this approach allows only rather crude estimate of the model parameters. In particular, it did not give us an estimate of the number of stem cells. This study further analyzes these data in light of the simple stem cell–niche model using a more sophisticated methodological framework. We present a full probabilistic model of the methylation patterns sampled from a crypt together with a Markov chain Monte Carlo (MCMC) algorithm allowing Bayesian inference of its parameters that include the number of stem cells, the depth of the genealogical tree (niche succession time), and the rate of the methylation/demethylation process. The fit of the model is assessed by comparing the observed values of several statistics summarizing the data with their posterior distribution under the model. We compare the estimates of the model parameters in either an unconstrained model or a model with short niche succession time.

## Results

### Polymorphic Methylation Patterns within the Crypts

In this study, we analyze methylation at nine CpG sites in a 77-bp locus within a CpG island upstream of the human biglycan (BGN) gene on chromosome X [15]. Our data consist of methylation patterns at loci randomly sampled from individual colon crypts from seven male patients between 40 and 87 years old.

**Figure 1:**
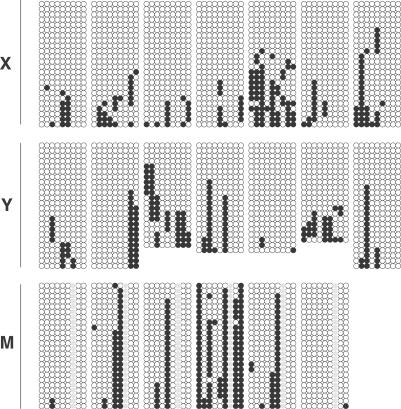
Sampled Methylation Patterns at the BGN Locus from Three Patients (Top to bottom) Patient X, 58 years old; patient Y, 81 years old; patient M, 87 years old. Each circle represents the status of one CpG site in one sequence (empty circle when unmethylated, filled circle when methylated). A group of nine circles aligned horizontally corresponds to a methylation pattern. The patterns from the same crypt form a block. Notice that the BGN locus of the 87 year-old patient contains only eight CpG sites due to a single nucleotide polymorphism (gray sites).

The total of 57 crypts studied here can be divided into eight-pattern and 24-pattern datasets. The eight-pattern data comprise the sequences of between five and 14 methylation patterns for 37 crypts isolated from five patients whom we described in a previous study [15]. The 24-pattern data are new, and correspond to the sequences of between 20 and 24 methylation patterns for another 20 crypts sampled from three patients (one patient is common to both the eight-pattern and 24-pattern datasets). The patterns sampled from these 20 crypts are shown in Figure 1.

All patients are males and therefore haploid for the BGN locus. This simplifies the modelling of the genealogy of the sampled sequences, as it ensures a one-to-one correspondence between sequences and cell lineages.

### Coalescent Model and Bayesian Methodology

Polymorphic methylation patterns arise from methylation and demethylation events that take place in the genealogy of the sampled cells. We propose a probabilistic model of the observed polymorphism in terms of biologically meaningful parameters that govern both the shape of this genealogy and the methylation process. Events taking place in stem cell lineages are believed to play a crucial role in the generation of the observed patterns, and we first present how the model accounts for the genealogy of those lineages along with their methylation process. We next explain how the model relates the sampled patterns to the patterns of the stem cell lineages.

The shape of the stem cell genealogy is described by two parameters, *N* and τ. *N* is the number of stem cells in a crypt and τ corresponds approximately to the average number of years before niche succession within a crypt. Formally, τ/2 is the average waiting time for the coalescence of a pair of lineages, whereas, from a biological point of view, τ/(*N*−1) is the average lifespan of a stem cell.

Methylation and demethylation in the stem cell genealogy is modelled as a point process whose rates ν are expressed in terms of expected number of events per CpG site in time τ (before niche succession time). In agreement with experimental observations [15], CpG sites at the BGN locus are modelled as initially unmethylated at the birth of the patient. Two models were compared for the somatic methylation/demethylation process. In a first model, we distinguish methylation and demethylation rates, but sites are independent. In a second, more complicated, model we allow for interactions between sites through rates that depend on the current level of methylation of the locus. We later refer to this model as the *context-dependent model*.

**Figure 2:**
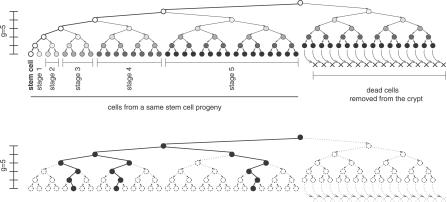
Modelling Differentiation Lineages (Upper panel) Shows the genealogical structure of a crypt subpopulation made of the progeny of the same stem cell. Differentiation process spans more than five rounds of cell divisions (g = 5), and differentiated cells are removed from the crypt when a new generation of differentiated cells arrives. Levels of gray ranging from white for the stem cell lineage to black for the differentiated cells indicate the different differentiation stages. (Lower panel) Illustrates the star-likeness of the genealogy of three cells randomly sampled from the progeny of the same stem cell. The lines of descent of these three cells are highlighted in black.

Sampled methylation patterns are related to those found in the stem cell lineages through three parameters, *g,* α, and ɛ. The parameter *g* can be interpreted as the number of cell cycles of the cell differentiation process. It describes the shape of the genealogy of cells sampled from the progeny of the same stem cell (illustrated in Figure 2). Higher values of *g* correspond to genealogies that tend to have longer terminal branches (and so are more star-like). The parameter α reflects the ratio between the amount of methylation and demethylation in a cell lineage during the cell differentiation process and in a stem cell lineage in time τ. More precisely, α = η/ν, where η is expressed in terms of the expected number of events in a single lineage during the few cell divisions of the differentiation process. The parameter ɛ corresponds to the rate of sequencing error per site per sequence.

Inference is carried out in a Bayesian framework using an MCMC algorithm designed to sample the posterior distribution of the parameters given the data. An uninformative prior is used. Parameters *N*, τ, *g,* and ɛ can take values in the intervals (2,50), (0.5,200), (5,10), and (0,1), respectively. Parameters ν, α can take any positive values. The inference framework was validated on simulated datasets.

### Assessment of Model Fitness

The methodology developed by Gelman et al. [18] served to assess the model adequacy. It consists in checking, through a set of statistics, the extent of the discrepancies between the data and datasets simulated with the model using parameters sampled from their posterior. Here data are summarized by the intercrypt average and standard deviation of five within-crypt statistics. These summary statistics are the number of distinct patterns, the number of polymorphic sites, the average pairwise distance between patterns (the average number of sites with distinct methylation status between pairs of patterns), the number of entirely unmethylated patterns, and the number of singletons (those patterns that appear only once in the crypt).

**Figure 3:**
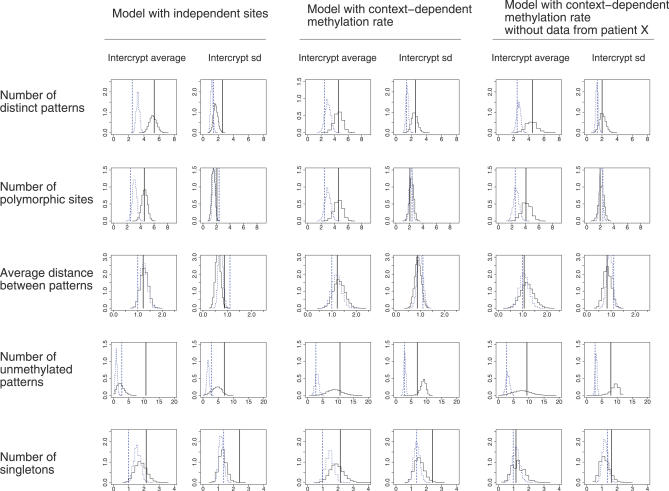
Assessment of Model Fitness (Left) Model with independent sites. (Middle) Model with context-dependent methylation rate. (Right) Model with context-dependent methylation rate without data from patient X. Five within-crypt statistics are examined (in rows). For each of these statistics, the observed intercrypt average and standard deviation are plotted (vertical lines) along with their expected distribution given the posterior of the parameters. Statistics that fall within their expected distributions indicate good fit of the model for the particular characteristics of the data measured by those statistics. The blue dashed lines show values computed for the eight-pattern-s (37 crypts) dataset, whereas black solid lines correspond to computations for the 24-pattern dataset (left and middle: 20 crypts; right: 13 crypts).

Expected distributions and empirical values of the five statistics are plotted in Figure 3. The fit of the model with context-dependent methylation rate is much better than the fit of the model with independent sites. This can be seen for instance in the average and standard deviation of the number of distinct patterns per crypt; in the standard deviation of the average distance between patterns of the same crypt; or in the average number of unmethylated patterns.

However, the model with a context-dependent methylation rate still shows some lack of fit. In particular, intercrypt variation in the number of singletons is higher than expected for the 24-pattern datasets. A detailed patient-by-patient analysis of the summary statistics (Table S1.1 in Protocol S1) reveals that this discrepancy may be explained by variability between patients: the observed intercrypt average of the number of singletons by patient falls above the upper limit 99% confidence interval of its expected distribution for patient X (4.0 singletons in average) and appears just at the lower limit of the 95% interval for patient X (0.86 singletons in average) (see Figure 1 for visual inspection).

**Figure 4:**
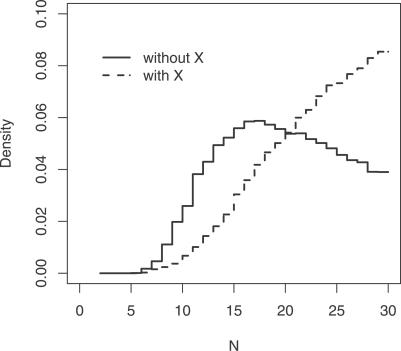
The Impact of Data from Patient X on the Posterior of *N* Comparison between posterior of *N* obtained with (dashed line) and without data from patient X (solid line).

This observation encouraged us to ignore data from patient X. The results then show a remarkable match between observed and simulated values (Figure 3, right, and Table S1.1 in Protocol S1). Such fit could not be achieved by exclusion of either patient Y or M (Table S1.2 in Protocol S1). Besides, data from patient X were found to shift the posterior of number of stem cells to higher values of *N,* probably due to the high number of singletons (Figure 4). The consequences of excluding either patient Y or patient M on the posterior of *N* were also explored (Figure S1.1 in Protocol S1), and patient X was found to impact the most on the posterior of *N*. Ignoring patient X actually softens our conclusions on the number of stem cells and can therefore be regarded as a conservative choice which further illustrates the relevance of a careful assessment of the model fit. We were also able to show that enhanced rates of methylation/demethylation are a possible hypothesis to explain the data from patient X (Figure S1.2 and Table S1.3 in Protocol S1).

### Posterior Suggests Many Stem Cells and Long Niche Succession Time

**Figure 5:**
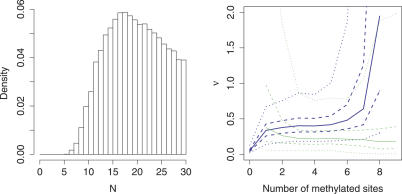
Posterior Distribution of *N* and ν Results are obtained using the model with context-dependent methylation rate and ignoring data from patient X. (Left) Posterior of the number of stem cells, *N*. (Right) Posterior of ν, the rate of the methylation process relative to the depth of the genealogy, depends on the number of methylated sites. Therefore, median (solid lines), first and third quartiles (dashed lines), and 95% confidence interval (dotted lines) of both the methylation rate (bold blue solid line) and demethylation rate (thin green solid line) are shown as a function of the number of methylated sites.

**Figure 6:**
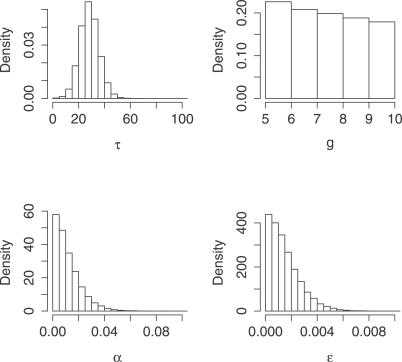
Posterior Distribution of τ, *g*, α, and ɛ Results are obtained using the model with context-dependent methylation rate and ignoring data from patient X. (Row 1) Reports the posteriors of the parameters τ, the average depth of the genealogy of the stem cells (in years), and *g,* the length of cell differentiation in terms of number of cell generations. These two parameters control, together with *N,* the shape of the genealogy. (Row 2) Reports the results for the parameters α and ɛ, responsible, together with ν, for the polymorphism given the genealogy: α is the relative amount of methylation/demethylation events in the *g* generations of cell differentiation compared with the number of events in a stem cell lineage up to the most recent common ancestor of the stem cell population; ɛ is the sequencing error rate.

The marginal posterior of each parameter of the model with context-dependent methylation rate obtained after removing data from patient X is shown in Figure 5 (*N* and ν) and Figure 6 (τ, *g*, α, and ɛ). The posterior distribution of *N*, τ, and *g* give a clear picture of the main features of the shape of the genealogy. The posterior distribution of the number of stem cells, *N,* reaches its mode between 15 and 20, gives little support for values of *N* below 8, and seems to exclude any value of *N* smaller than 6. The posterior distribution of τ suggests that the actual value of this parameter, which corresponds approximately to the average time before the stem cell population finds its most recent common ancestor, is located between 15 and 40 years. Finally, there seems to be very little information on parameter *g* that accounts for the shape of the genealogy of the cells sampled from the progeny of the same stem cell: the posterior distribution of *g* closely matches its continuous uniform prior on (5,10).

The posterior distributions of the parameters that explain the polymorphism given the genealogy are also enlightening. Parameter ν reveals synergistic methylation/demethylation across the sites of the BGN locus. The methylation rate is found to be highly dependent on the number of already methylated sites. The rate is very low when no sites are methylated and shows a more than fivefold increase when one site is already methylated (the median of ν moves from 0.05 to 0.35 methylation events per site in time τ). It is then relatively constant up to seven methylated sites and then increases again. In contrast, demethylation dynamics does not seem to depend on the current level of methylation. The posterior distribution of the parameter α suggests that methylation/demethylation events during cell differentiation contribute little to the observed polymorphism: its density decreases sharply between zero and 0.04, where it becomes negligible. Finally, the posterior distribution of ɛ indicates that sequencing errors are extremely rare (rate smaller than 0.004 and more likely between zero and 0.002).

It is worth emphasizing the overall coherence of the picture that emerges of this posterior inference: the genealogical trees of the stem cell lineages up to their most recent common ancestor are rather deep (high τ), and most methylation/demethylation events occur in those lineages (small α). Furthermore, the value of α seems compatible with the same probability of methylation/demethylation events per cell cycle during differentiation and in the stem cell lineages. A value of τ of about 20 years suggests about 1,000 rounds of cell divisions in the stem cell lineages before these lineages find their most recent common ancestor (stem cells are thought to divide about weekly in the human colon [6,11]), whereas the number of rounds of cell divisions during differentiation is certainly smaller than ten. Therefore, we expect a value of α smaller than 0.01 = 10/1,000.

The comparison with the posterior obtained from the full dataset including patient X (Figures S1 and S2) reveals that the data from patient X not only impact on the posterior of *N* but also to a lesser extent on the distribution of α, ɛ, and the demethylation rates of hypomethylated sequences. In each case, patient X shifts the distribution toward higher values of the parameters. Inspection of the posterior obtained when considering only the 24-pattern data without patient X (Figures S3 and S4) indicates that most of the information on *N*, *g*, α, and ɛ is contained in the 24-pattern datasets while the eight-pattern datasets greatly contributed to the information on τ and to a lesser extent to the information on ν.

### Many Stem Cells versus Methylation Changes During Cell Differentiation

**Figure 7:**
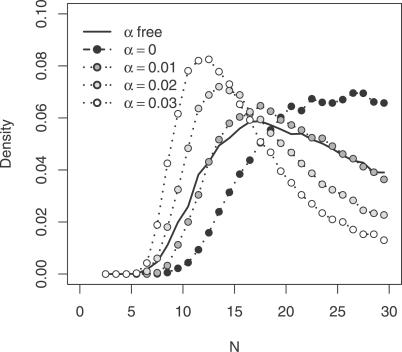
Posterior Correlation between *N* and α The posterior of *N* subject to the constraint α = 0, 0.01, 0.02, or 0.03 (dotted lines) are compared with the posterior of *N* without those constraints (solid line).

Unconstrained posterior inference suggests that the high level of intracrypt polymorphism is due to the existence of many stem cells in each crypt (high *N*). An alternative explanation for this high level of polymorphism could be a significant amount of methylation/demethylation events taking place in differentiation lineages (high α). Although this hypothesis does not receive support from our analysis, a closer look at the posterior reveals a negative correlation between *N* and α shown in Figure 7. The posterior of *N* for values of α below 0.01 virtually excludes values of *N* below ten, but a number of stem cells below ten becomes likely when α increases. These observations allow a better interpretation of the posterior of α and *N* obtained in the unconstrained analysis: a number of stem cells between eight and ten is unlikely but may be compatible with the data if α > 0.01. In the context of a niche succession time of 20 years, this would indicate that the rate of methylation/demethylation is enhanced during cell differentiation.

**Figure 8:**
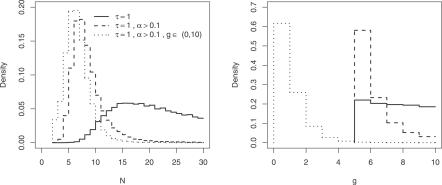
Posteriors of *N* and *g* in Models with Short Niche Succession Time Three models that assume a niche succession time of about one year (τ = 1) are compared: same model as before but with τ = 1 (plain line); τ = 1 and high level of methylation/demethylation during cell differentiation (α > 0.1, dashed line); τ = 1, α > 0.1, without imposing a star-like genealogy for differentiation lineages (0 < *g* < 10, dotted line).

The model and its associated inference procedure provide an invaluable tool to further explore the hypothesis of a high amount of pattern changes during cell differentiation. A relatively high value of α is particularly realistic in a scenario with short niche succession time (small τ). As an illustration, we investigate here what could be the consequence of a niche succession time of about one year (τ = 1). We can see in Figure 8 that setting the parameter τ to 1 has no impact on the posterior of *N*. We also found that the constraint τ = 1 has no impact on the estimate of α (unpublished data) and *g* (Figure 8, right panel). From a biological point of view, however, a short niche succession time does not seem compatible with a very small value of α. Indeed, a τ of one year suggests that niche succession may be reached after only 50 cell cycles, compared with a minimum of five cell cycles for cell differentiation. We thus expect α greater than 0.1 = 5/50. The posterior of *N* was therefore investigated subject to this constraint. Results are presented in Figure 8 and reveal the deep impact of such a high value of α on the posterior of *N,* which now indicates a value of *N* between four and 12, whereas the value of *g* is close to five.

This impact of α prompted us to understand why the data do not support a high value of α under our model assumptions. The posterior of *g* concentrated close to its lower bound suggest that *g* > 5 might be incompatible with α > 0.1. This hypothesis is confirmed after examination of the posterior of *g* when allowing *g* to take values smaller than five (see Figure 8, right panel). The density decreases sharply between zero and three and excludes values of *g* higher than four. Data seem, therefore, incompatible with a scenario where a significant fraction of the methylation/demethylation events take place during cell differentiation across star-like genealogies.

## Discussion

The analysis of methylation patterns is a promising approach to investigate the structure of cell populations in an organism [15,19–21]. In a stem cell–niche scenario, sampled methylation patterns are the stochastic outcomes of a complex interplay between niche structural features such as the number of stem cells within a niche and the niche succession time, the methylation/demethylation process, and the randomness due to the sampling. As a consequence, methylation pattern studies can reveal niche characteristics but also require appropriate statistical methods. The analysis of methylation patterns sampled from colon crypts is a prototype of such a study. Previous analyzes were based on forward simulations of the whole cell content of the crypt and subsequent comparisons between simulated and experimental data using a few statistics as a proxy to summarize the data (number of distinct patterns per crypt, average methylation, and intracrypt distance). In this paper we develop an alternative inference framework based on likelihood computations that make full use of the data rather than making use of the values of a few summary statistics.

Our assumptions about the biological mechanisms underlying the methylation patterns we observe are essentially the same as in previous works but we reformulate the model backward in time as a coalescent process. Coalescent modelling is a starting point for carrying out likelihood inference that makes use of the full data. It is also interesting by itself as it permits direct simulations of the small part of the whole crypt history that is relevant for explaining observed samples of methylation patterns, the history of the sampled cell lineages.

We developed an MCMC algorithm that allows inference of all the parameters of the model. It is worth emphasizing that the inference relies on a number of model assumptions that we can summarize in four points: (1) the stochastic process generating the methylation pattern we sample is the same in all crypts, except for the age of the crypts, which differ among patients; (2) the number of stem cells is stable and the loss of a stem cell is compensated by the symmetric division of one of the other stem cells. This justifies modelling the stem cell genealogy as a simple coalescent process (this supposes, for instance, that stem cell losses are not compensated by new stem cells originating from another layer of stem cells); (3) the genealogy of differentiation is star-like when branch lengths are measured through the accumulation of methylation/demethylation events; (4) appropriate modelling of methylation/demethylation can be achieved using a simple continuous-time point process across lineages.

These assumptions are intended to be as reasonable as possible, and the demonstration that the data are compatible with this simple model is one merit of this work. Careful assessment of the model fit, nevertheless, revealed the need for partial relaxation of hypotheses (1) and (4). Data from patient X were ignored owing to too high a number of singletons, and a context-dependent model allowing methylation and demethylation rates to vary with the number of already methylated sites was introduced. In the future, additional data may reveal the need for further refinement of the model, and we can envision modelling parameter variation across crypts and patients or introducing distinct parameters for the methylation processes in stem cell and differentiation lineages.

The need for a context-dependent model of the methylation process brings another piece of evidence for synergistic methylation processes that are also supported by a number of experimental studies. However, this seems to be the first study that directly assesses those effects on the basis of their long-term consequences such as the transition between hypomethylated and hypermethylated sequences that translate into the coexistence of both types of sequences within the crypt. These effects are likely to rely on interactions between maintenance methyl-transferase (Dnmt1) and de-novo methyl-transferase (Dnmt3a/b). It is believed that de-novo methylation by Dnmt3a/b (de-novo methyl-transferase) is stimulated by Dnmt1 (maintenance methyl-transferase) acting to maintain the methylation status through the methylation of the newly synthesized DNA strand at hemimethylated sites after replication [22,23]. This mechanism could explain the increase in the rate of methylation while the rate of demethylation remains stable.

Development of models that can effectively account for both dependencies between sites and variation in methylation/demethylation rates across CpG sites will be an interesting challenge. Our results show that the average distance between patterns and the number of distinct patterns unambiguously call for context-dependent effects. These statistics are known to be important indicators of the effective population size in population genetics [24,25]. However, examination of the level of methylation at each site of the BGN locus suggests the existence of some differences between sites [15].

The main challenge in the estimation of the number of stem cells from methylation patterns is to distinguish polymorphism due to methylation/demethylation events in stem-cell lineages and the polymorphism due to events during cell differentiation. The framework proposed here allows us to address this issue in a quantitative manner. The results suggest that the methylation changes in differentiation lineages are rare while the number of stem cells is higher than eight and reaches its posterior mode between 15 and 20. The small contribution of the events taking place in differentiation lineages to the diversity of methylation patterns is coherent with our estimate of a relatively long niche succession time of more than 15 years. This “high *N*–high τ” scenario can account for the rapid decline in the number of partially mutant crypts reported by Campbell et al. [13], as it is compatible with a short life of individual stem cells (average τ/(*N*−1)). The almost negligible amount of methylation events in the few cell divisions of the differentiation process is also in agreement with small rates of methylation/demethylation experimentally measured in some human cell lineages around 0.001 change/site/generation [26–28], but contrasts with the high rates found in other lineages or other loci where errors in the replication of the methylation pattern attain 0.01 to 0.15 change/site/generation [22,28,29,30]. Changes are believed to occur when sites are hemimethylated at the time of DNA replication after either de novo or incomplete maintenance methylation. In the future, experimental assessment of the frequency of those hemimethylated sites by double-strand DNA methylation pattern sequencing [28] in cells sampled from the crypt may help to further calibrate the amount of methylation changes in the differentiation lineages.

Sequence redundancy artifacts caused by bisulfite treatment and PCR amplification have been detected in the context of bisulfite sequencing, although in a distinct experimental setting [31]. In this study we limited our analysis to a short 77-bp locus, sampled a relatively small number of times (fewer than 24) compared with the number of copies available from the biological sample (more than 1,000), and amplified in four independent PCRs. All these precautions certainly decrease the chances of artifacts [32,33]. In addition, we have previously reported pattern redundancy at the BGN locus between samples from both parts of bisected crypts [15], and this suggests that a substantial fraction of the pattern redundancy reported here reflects genuine biological redundancy. Finally, our main biological conclusions are probably robust against limited sequence redundancy artifacts. Little is known about those artifacts, but intuitively we think they are more likely to decrease than to increase the apparent number of stem cells, and they can hardly be invoked in place of synergistic methylation to explain the coexistence within the same crypts of a substantial proportion of fully unmethylated patterns and a diversity of related methylation patterns. We nevertheless acknowledge the need for better verified data; molecular barcoding will be the method of choice for this purpose [29,31].

The inference framework proposed in this study will be an invaluable tool to address questions related to the design of future experiments. We could, for instance, wonder whether it will be better to assess more cells or to get higher resolution in the description of the cell lineages by sequencing more CpG sites. As sequencing longer patterns could prove technically hard, an intermediate route could consist in sequencing additional loci sampled from the same crypt but from independent cells. Our approach could rather easily be extended to handle this kind of multilocus data as well as diploid locus data. We also envision the development of less computationally demanding inference methods based on summary statistics, for which our work provides both a simulation tool and a benchmark.

## Materials and Methods

### Colonic assays.

Individual crypts were isolated from fresh colectomy specimens, and, after extracting the crypt DNA content, unmethylated cytosines were converted into uracil by bisulfite treatment. The bisulfite-treated DNA was further amplified by quantitative PCR, and the BGN locus of a relatively small number of molecules (five to 24) was sampled and sequenced. Patients had colectomies for adenocarcinoma, but the normal colon crypts examined in this study were taken at least 10 cm away from the tumors, and are unlikely to be directly involved with tumorigenesis. Details of the experimental protocol can be found in [15].

### Modelling stem cell lineages.

We model the size of the stem cell population in a crypt as identical in all crypts, in all patients, and kept at a constant value *N* throughout the life of the patients. A stem cell is said to die if it gives birth to two cells committed to differentiation. The death of one stem cell is assumed to be instantly compensated by a symmetric division of one of the remaining stem cells (chosen at random) that produces two stem cells. We model the lifespan of a stem cell as an exponential random variable with rate γ (mean 1/γ). Under these assumptions, when looking backward in time the stem cell genealogy follows a particular coalescent process [34], a version of the Moran model [35]. The waiting time for the coalescence of two stem cell lineages is an exponential random variable with rate 2γ/(*N*−1). When considering *k* lineages, the waiting time for the first coalescence event of two lineages is exponential with rate γ*k*(*k*−1)/(*N*−1). Furthermore, the two lineages that coalesce are chosen at random with probability 2/*k*(*k*−1). This representation would be clearly inadequate if used for the entire cell population of the colon crypt, as it would not account for the very different behavior of the stem cells and differentiating cells [36]. We explain later how our model accounts for the properties of the lineages of differentiating cells.

In agreement with our previous experimental observations [15], all the CpG sites that we analyze here are supposed unmethylated at the birth of the patient, and the observed methylation patterns are supposed to result from methylation and demethylation events that took place across the genealogy of the sampled cells. In a first simple model, we assume that every CpG site evolves independently at the same constant rate. Such a model has two parameters μ = (μ_+_,μ_−_), where μ_+_ is the rate of methylation per site and μ_−_ is the rate of demethylation per site.

We also introduce a second, more sophisticated, model that accounts for context-dependent effects on the evolution rate. In this model, the rate of methylation and demethylation is allowed to vary with the number of already methylated sites. To avoid introducing a number of parameters as large as twice the number of CpG sites, we consider only four sets of methylation/demethylation rates that apply on four distinct ranges of numbers of methylated sites. Range boundaries will be estimated. In the general case, the use of models where sites do not evolve independently requires prohibitively heavy computation. However, when all sites are supposed to evolve according to the same model, we show how to take advantage of the model symmetry to considerably speed up the computations (see Protocol S2, section 1, “Calculations in the context-dependent model”). To our knowledge, it is the first use of this context-dependent model for modelling biological sequences.

### Indirect sampling of the stem cell lineages.

Methylation patterns sampled within the crypt are not directly sampled from stem cell lineages. Most of them come from differentiated and differentiating cells that are the product of the few rounds of cell division that take place during cell differentiation. The next two paragraphs explain how our model relates the sampled methylation patterns to the stem cell methylation patterns.

We suppose that the cell content of the crypt is composed of *N* equal-sized subpopulations, each one corresponding to the progeny of one of the stem cells. As we know that differentiated cells are short-lived and that the differentiation process spans only a few generations of cells, it is attractive to assume that all cells sampled from the same subpopulation share the methylation pattern of their most recent common stem cell ancestor. Under this hypothesis, we do not need to keep track of the precise genealogical process that goes back in time from the sampled cell to the most recent ancestral stem cell. All we need is to model which sampled cells come from the same stem cell lineage. A model of sampling with replacement is fairly reasonable for this purpose as we believe that subpopulations are large compared with the number of sampled patterns and DNA is amplified by PCR before sequencing. In this model, when we sample *n* sequences, we actually sample a random number *M ≤ N* of stem cell lineages. The probability mass function for Γ, the random variable that defines *M* and the partitioning of the *n* sequences into *M* groups, is given by

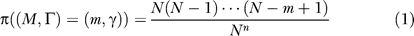



Preliminary numerical experiments suggested that estimates of *N* based on this simple model would be misleading even for a limited amount of methylation/demethylation during cell differentiation. Therefore, we preferred a more general model that can account for methylation and demethylation during cell differentiation. For this purpose, the model needs to describe the genealogy of the cells sampled from the same subpopulation (Equation 1) back in time until its ancestral stem cell lineage. Under our model, the genealogy of *q* cells sampled from the same subpopulation results from a coalescent process between random pairs of lineages whose times (*s_q_*,*s_q_*_−1_,…,*s*_2_) are drawn according to the probability density function

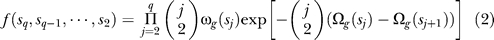
where 0 ≤ *s_q_* ≤ *s_q_*_−1_ ≤ … ≤ *s*_2_ <1 and *ω_g_* is the first derivative of Ω*_g_*, an integrated rate function defined by Ω*_g_*(*t*) = −log((2*^g^*−2*^gt^*)/(2*^g^*−1)). The parameter *g* controls the star-likeness of the genealogy of the subsample back in time until the lineages of all the differentiated cells of the crypt are stem cell lineages. The form of the integrated rate function Ω*_g_* is justified in Protocol S2 (section 2, “Genealogy of cells sampled from the progeny of the same cell”) as a continuous-time approximation of the discrete genealogical process of the subsample under a simplistic model where each subpopulation is the result of *g* rounds of cell divisions. The timescale of this process is expressed in arbitrary units that we do not try to compare with the timescale of the genealogy of the stem cell lineages. Rather, the methylation/demethylation process across the branches of this genealogy has its own rate denoted η = (η_+_,η_−_) that is proportional to μ = (μ_+_,μ_−_), the rate of the methylation/demethylation process in the stem cell lineages.

Possible sequencing errors were also accounted for through a parameter ɛ which corresponds to the probability of error at one CpG site of a methylation pattern.

### Parametrization and priors.

Although we tried to introduce as few parameters as possible in our model, any estimate of its parameters will clearly be associated with a relatively high level of uncertainty due to the limited amount of data. The Bayesian statistical framework provides a straightforward approach to account for this uncertainty by allowing us to compute the posterior distribution of the parameters given the available data. However, the choices of a parametrization and a prior distribution of the parameters are important issues in the Bayesian context. Without reliable a priori information, it is natural to look for an uninformative prior. To our knowledge, current Bayesian methodology provides few guidelines that may be useful in the context of our study. The approach we decided to adopt consists of finding a parametrization that minimizes the dependencies between the parameters according to the posterior distribution. Several motivations justify this approach: it makes reasonable the use of independent priors for each parameter, it facilitates the interpretation of the posteriors, and it helps in designing efficient MCMC algorithms to explore the posterior.

We chose a uniform distribution for the number of stem cells, *N,* which is the primary focus of our interest. *N* is also the only parameter that impacts on *M,* the random number of stem cell lineages sampled in a particular crypt.

The speed of the coalescent process modelling the stem cells genealogy is a function of the ratio γ/(*N−*1). We denote the inverse of this ratio by τ = (*N−*1)/γ, which corresponds approximately to the expected number of years before the entire stem cell population finds a common ancestor (without considering the truncating effect of the birth on this coalescent), and chose a uniform prior on (0.5,200) for it. This seems a better choice than direct modelling of γ, the expected lifespan of a stem cell, as when *n* is small enough compared with *N* then *M* is equal or close to *n* and the only effects we observe are those of the ratio γ/(*N*−1). Under these conditions, the posterior distribution of γ (but not τ) will be highly correlated with that of *N*. The parameter *g* that accounts for the star-likeness of the genealogy of the cells sampled from the progeny of the same stem cell was chosen from a uniform density on the interval (5,10).

Concerning the methylation process, it is worth mentioning that the rate of the coalescent and the overall speed of the methylation process are distinguishable only if the methylation pattern in the most recent common ancestor of the stem cell population does not follow the stationary distribution of the methylation process. When the stationary distribution of the methylation process is reached in this ancestor, the data carry information only about the relative speed of the methylation compared with the depth of the genealogical tree (μτ). As a consequence, μ and τ can be highly correlated under their joint posterior while ν = μτ and τ would be relatively independent. We therefore preferred a prior that models ν independent of τ rather than μ independent of τ. Looking for an uninformative prior on ν, we chose a log-normal distribution such that log(ν) is normally distributed with mean zero and standard deviation σ. The potential difficulty of the choice of σ was bypassed by modelling σ as an exponential random variable with mean one (a strategy known as hyper-prior modelling). The rate η of the methylation process relative to the arbitrary timescale of the genealogy of the differentiation lineages was chosen as η = αν where α follows an exponential distribution with mean one. The parameter α corresponds to the relative amount of methylation/demethylation events taking place before a sampled cell finds its stem cell ancestor, compared with the number of events occurring in the lineage of this stem cell up to the most recent common stem cell ancestor.

Finally, we modelled the probability of sequencing errors, ɛ, as a continuous uniform on (0,1).

### MCMC algorithm and software.

The posterior distribution of the parameters has been investigated using an MCMC algorithm [37]. Denoting ***X*** the observed methylation patterns, the purpose of the algorithm is to sample from the joint posterior distribution of the parameters θ = (*N*,τ,*g*,σ,ν,α,ɛ) given ***X*** (we use bold fonts to emphasize where there is a random variable per crypt analyzed). An MCMC algorithm creates a sample of dependent realizations from the target distribution by updating in turn the components of θ, each update preserving the target distribution. For practical reasons, the MCMC algorithm samples an augmented space much larger than θ. It consists of (θ,Λ,***Y***), where Λ denotes the genealogies of the methylation patterns (topology and coalescent times) and ***Y*** stands for the methylation patterns in the nodes of Λ.

Updating *N* in our model is difficult and we propose an original strategy to solve the problem. As explained in Protocol S2 (section 3, “A slightly modified model for the number of stem cells”), our approach consists of embedding our model in a slightly more general model that allows *N* to differ by one between crypts. Another challenging task of the algorithm is to explore the huge space of possible genealogies Λ. This is performed efficiently using the “branch-swapping” strategy introduced by Wilson and Balding [38]. In this scheme, the sequences ***Y*** at the nodes of Λ serve to propose relevant modifications of the genealogy. Further details of the algorithm are given in Protocol S2 (section 4, MCMC algorithm).

We obtained all the results presented here by running the algorithm for 5,000,000 iterations and recording (*N*,τ,*g*,ν,σ,α,ɛ,Λ,***Y***) every 100 iterations once past the first 500,000 iterations. Systematic visual checking of the samples did not reveal convergence problems. Each run of the MCMC algorithm takes approximately ten days on a 3-GHz Intel Pentium processor when the model with context-dependent methylation rate is used.

Software and data can be downloaded at http://genome.jouy.inra.fr/~pnicolas/mcmcniche/.

### Estimating the number of stem cells on simulated datasets.

Bayesian methodology ensures that posterior distributions are meaningful “on average” for values of the parameters drawn from their prior, although the data are generally compatible only with a small fraction of the parameter values allowed by the prior. It is therefore a good idea to check that the prior gives relevant results for combinations of parameters having some level of compatibility with the data.

We validated our inference framework on ten synthetic datasets. Five were simulated with *N* = 6, whereas the other five were simulated with *N* = 24. Two sets of values for τ, ν, α, *g,* and ɛ were chosen after running the MCMC algorithm with *N* constrained either to six or 24. Both sets of parameters are given in Protocol S2 (section 5, “Parameters used to generate simulated datasets”). They differ mostly in the value of α, which reflects the relative contribution to the polymorphism of the methylation/demethylation events taking place during cell differentiation compared with those occurring in stem cell lineages. The six and 24 stem cell datasets were simulated with α = 0.082 and α = 0.018, respectively.

**Figure 9:**
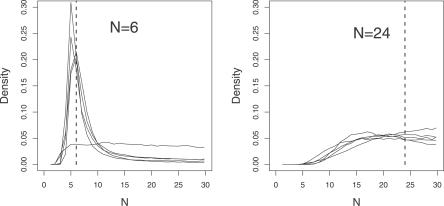
Posterior Distribution of *N* for Simulated Datasets On the left side *N =* 6, whereas on the right side *N* = 24.

Posterior distributions were able to distinguish between both series of datasets (Figure 9). For all but one dataset simulated with *N* = 6, the posterior shows a clear peak around five or six while the last dataset is less informative as the posterior gives a similar support for any value of *N* greater than five. On the other hand, posterior distributions obtained on datasets simulated with *N* = 24 were all found to increase slowly between *N* = 5 and *N* = 15 and to be relatively flat for *N* greater than 15.

Two kinds of effects combine to explain that posteriors of *N* obtained for large *N* are flatter than posteriors found for small *N*. First, the posterior mechanically becomes flatter as *N* increases because models with neighbor values of *N* tend to look more and more alike. Second and less obvious, the data carry an amount of information on *N* that is limited by the number of cells sampled from each crypt, as all the information on *N* comes from the fact that some patterns are sampled from the progeny of the same stem cell (see Equation 2). When *N* becomes large compared with the number of cells sampled in each crypt, each cell tends to belong to the progeny of a different stem cell, and at best we may be able to say that *N* is large compared with the number of patterns sampled.

## Supporting Information

Figure S1 Posterior Distribution of the Number of Stem Cells and the Methylation and Demethylation Rates Obtained on the Full Data (with Patient X) Using the Model with Context-Dependent Methylation Rate (6 KB PDF)

Figure S2 Posterior Distribution of the Other Parameters Obtained on the Full Data (with Patient X) Using the Model with Context-Dependent Methylation Rate (6 KB PDF)

Figure S3 Posterior Distribution of the Number of Stem Cells and the Methylation and Demethylation Rates Obtained on the 24-Pattern Data (without Patient X) Using the Model with Context-Dependent Methylation Rate (6 KB PDF)

Figure S4 Posterior Distribution of the Other Parameters Obtained on the 24-Pattern Data (without Patient X) Using the Model with Context-Dependent Methylation Rate (6 KB PDF)

Protocol S1 Supporting Information on the Exclusion of Data from Patient X (67 KB PDF)

Protocol S2 Supporting Materials and Methods (79 KB PDF)

## Abbreviations

BGN: human biglycan
MCMC: Markov chain Monte Carlo

## References

[pcbi-0030028-b001] Ohlstein B, Kai T, Decotto E, Spradling A (2004). The stem cell niche: Theme and variations. Curr Opin Cell Biol.

[pcbi-0030028-b002] Li L, Xie T (2005). Stem cell niche: Structure and function. Annu Rev Cell Dev Biol.

[pcbi-0030028-b003] Yamashita YM, Fuller MT, Jones DL (2005). Signaling in stem cell niches: Lessons from the *Drosophila* germline. J Cell Sci.

[pcbi-0030028-b004] Xie T, Spradling AC (2000). A niche maintaining germ line stem cells in the *Drosophila* ovary. Science.

[pcbi-0030028-b005] Bach SP, Renehan AG, Potten CS (2000). Stem cells: The intestinal stem cell as a paradigm. Carcinogenesis.

[pcbi-0030028-b006] Potten CS, Booth C, Hargreaves D (2003). The small intestine as a model for evaluating adult tissue stem cell drug targets. Cell Prolif.

[pcbi-0030028-b007] Leedham SJ, Brittan M, McDonald SA, Wright NA (2005). Intestinal stem cells. J Cell Mol Med.

[pcbi-0030028-b008] Potten CS, Booth C, Tudor GL, Booth D, Brady G (2003). Identification of a putative intestinal stem cell and early lineage marker; musashi-1. Differentiation.

[pcbi-0030028-b009] Kaur P, Potten CS (1986). Cell migration velocities in the crypts of the small intestine after cytotoxic insult are not dependent on mitotic activity. Cell Tissue Kinet.

[pcbi-0030028-b010] Qiu JM, Roberts SA, Potten CS (1994). Cell migration in the small and large bowel shows a strong circadian rhythm. Epithelial Cell Biol.

[pcbi-0030028-b011] Potten C, Kellett M, Roberts S, Rew D, Wilson G (1982). Measurement of in vivo proliferation in human colorectal mucosa using bromodeoxyuridine. Gut.

[pcbi-0030028-b012] Potten CS, Owen G, Booth D (2002). Intestinal stem cells protect their genome by selective segragation of template DNA strand. J Cell Sci.

[pcbi-0030028-b013] Campbell F, Williams GT, Appleton MA, Dixon MF, Harris M (1996). Post-irradiation somatic mutation and clonal stabilisation time in the human colon. Gut.

[pcbi-0030028-b014] Roberts SA, Hendry JH, Potten CS (2003). Intestinal crypts clonogens: A new interpretation of radiation survival curve shape and clonogenic cell number. Cell Prolif.

[pcbi-0030028-b015] Yatabe Y, Tavaré S, Shibata D (2001). Investigating stem cells in human colon by using methylation patterns. Proc Natl Acad Sci U S A.

[pcbi-0030028-b016] Kim JY, Siegmund KD, Tavaré S, Shibata D (2005). Age-related human small intestine methylation: Evidence for stem cell niches. BMC Med.

[pcbi-0030028-b017] Pfeifer GP, Steigerwald SD, Hansen RS, Gartler SM, Riggs AD (1990). Polymerase chain reaction-aided genomic sequencing of an X chromosome–linked CpG island: Methylation patterns suggest clonal inheritance, CpG site autonomy, and an explanation of activity state stability. Proc Natl Acad Sci U S A.

[pcbi-0030028-b018] Gelman A, Meng XL, Stern H (1996). Posterior predictive assessment of model fitness via realized discrepancies. Statistica Sinica.

[pcbi-0030028-b019] Kim JY, Tavaré S, Shibata D (2005). Counting human somatic cell replications: Methylation mirrors endometrial stem cell divisions. Proc Natl Acad Sci U S A.

[pcbi-0030028-b020] Kim JY, Tavaré S, Shibata D (2006). Human hair genealogies and stem cell latency. BMC Biol.

[pcbi-0030028-b021] Shibata D, Tavaré S (2006). Counting divisions in a human somatic cell tree: How, what and why?. Cell Cycle.

[pcbi-0030028-b022] Liang G, Chan MF, Tomigahara Y, Tsai YC, Gonzales FA (2002). Cooperativity between DNA methyltransferases in the maintenance methylation of repetitive elements. Mol Cell Biol.

[pcbi-0030028-b023] Kim GD, Ni J, Kelesoglu N, Roberts RJ, Pradhan S (2002). Cooperation and communication between the human maintenance and de novo DNA (cytosine-5) methyltransferases. EMBO J.

[pcbi-0030028-b024] Ewens W (1972). The sampling theory of selectively neutral alleles. Theor Popul Biol.

[pcbi-0030028-b025] Tajima F (1983). Evolutionary relationship of DNA sequences in finite populations. Genetics.

[pcbi-0030028-b026] Ushijima T, Watanabe N, Okochi E, Kaneda A, Sugimura T (2003). Fidelity of the methylation pattern and its variation in the genome. Genome Res.

[pcbi-0030028-b027] Ushijima T, Watanabe N, Shimizu K, Miyamoto K, Sugimura T (2005). Decreased fidelity in replicating cpg methylation patterns in cancer cells. Cancer Res.

[pcbi-0030028-b028] Laird CD, Pleasant ND, Clark AD, Sneeden JL, Hassan KM (2004). Hairpin-bisulfite PCR: Assessing epigenetic methylation patterns on complementary strands of individual DNA molecules. Proc Natl Acad Sci U S A.

[pcbi-0030028-b029] Genereux DP, Miner BE, Bergstrom CT, Laird CD (2005). A population-epigenetic model to infer site-specific methylation rates from double-stranded DNA methylation patterns. Proc Natl Acad Sci U S A.

[pcbi-0030028-b030] Sontag LB, Lorincz MC, Luebeck EG (2006). Dynamics, stability and inheritance of somatic DNA methylation imprints. J Theor Biol.

[pcbi-0030028-b031] Miner B, Stoger R, Burden A, Laird C, Hansen R (2004). Molecular barcodes detect redundancy and contamination in hairpin-bisulfite PCR. Nucleic Acids Res.

[pcbi-0030028-b032] Warnecke P, Stirzaker C, Song J, Grunau C, Melki J (2002). Identification and resolution of artifacts in bisulfite sequencing. Methods.

[pcbi-0030028-b033] Millar D, Warnecke P, Melki J, Clark S (2002). Methylation sequencing from limiting DNA: Embryonic, fixed, and microdissected cells. Methods.

[pcbi-0030028-b034] Kingman JFC (1982). On the genealogy of large populations. J Appl Prob.

[pcbi-0030028-b035] Moran PAP (1962). The statistical processes of evolutionary theory.

[pcbi-0030028-b036] Nowak MA, Michor F, Iwasa Y (2003). The linear process of somatic evolution. Proc Natl Acad Sci U S A.

[pcbi-0030028-b037] Gilks WR, Richardson S, Spiegelhalter DJ (1997). Markov chain Monte Carlo in practice. Chapman and Hall.

[pcbi-0030028-b038] Wilson IJ, Balding DJ (1998). Genealogical inference from microsatellite data. Genetics.

