# Supplementary material for: The Stem Cell Population of the Human Colon Crypt: Analysis via Methylation Patterns
Source: PLoS Comput Biol. 2007 Mar 2;3(3):e28. doi: 10.1371/journal.pcbi.0030028 (PMC1808490; doi:10.1371/journal.pcbi.0030028)
Supplement: Supplementary file 1 [file pcbi.0030028.sd001.pdf]

Protocol S1. Supporting information related to the exclusion of data from patient X.

S1.1 Summary statistics

Model adequacy was investigated separately for each patient. The empirical values of the five summary statistics were compared with their expected distributions given parameters sampled from the posterior obtained on different data sets (full data, data without either X, Y or M). Results are reported in Table S1.1.

Amongst all statistics and all patients, the average number of singletons in crypts from patient X stands out with by far the most pronounced departure from its expected distribution given the full data ($\mathbb{P}(X_{pred} \leq X_{obs}) = 0.003$). As we mentioned in the main body of the manuscript, the number of singletons in crypts from patient Y is also unexpected ($\mathbb{P}(X_{pred} \leq X_{obs}) = 0.975$) but to a much lesser extent. This was our primary motivation for ignoring the data from patient X.

It can also be seen that when the parameters are estimated without the data from patient X, the number of singletons observed in crypts from patient Y becomes compatible with the parameters. Conversely, neither the exclusion of patient Y or M renders the data from patient X compatible with the rest of the data (Table S1.1).

Table S1.2 reports the consequences of ignoring patient X, Y or M on model adequacy assessed through the averages and the standard deviations of the five summary statistics computed across the crypts from the different patients. As in the patient-by-patient analysis, these results on the standard deviation of the number of singletons show that exclusion of patient X makes the observed value of the statistic compatible with its expected distribution. Exclusion of patient Y or patient M does not achieve this result.

		obs[a]		p_{MXY}[b]		p_{MY}		p_{MX}		p_{XY}	
Statistic	set[c]	ave.[d]	sd[e]	ave.	sd	ave.	sd	ave.	sd	ave.	sd
Distinct patterns	I	2.14	1.07	0.810	0.724	0.654	0.634	0.818	0.741	0.836	0.724
	D	2.14	0.90	0.746	0.813	0.587	0.749	0.754	0.823	0.776	0.818
	F	3.00	1.50	0.591	0.604	0.346	0.448	0.614	0.617	0.636	0.589
	H	2.67	2.25	0.824	0.027	0.673	0.012	0.831	0.033	0.848	0.029
	M8	2.78	1.92	0.800	0.064	0.603	0.028	0.804	0.070	0.839	0.056
	X	7.00	2.83	0.035	0.276	—	—	0.068	0.346	0.042	0.258
	Y	4.57	1.51	0.747	0.918	0.422	0.842	—	—	0.785	0.900
	M24	4.33	2.66	0.772	0.284	0.474	0.130	0.823	0.317	—	—
Polym. sites	I	1.43	1.27	0.879	0.900	0.781	0.865	0.879	0.903	0.920	0.932
	D	2.43	3.10	0.404	0.024	0.277	0.014	0.398	0.018	0.507	0.031
	F	3.33	2.69	0.372	0.213	0.201	0.139	0.342	0.165	0.493	0.244
	H	2.83	3.31	0.687	0.032	0.533	0.020	0.654	0.023	0.771	0.041
	M8	2.78	1.92	0.926	0.510	0.849	0.434	0.915	0.431	0.953	0.516
	X	5.43	2.07	0.139	0.732	—	—	0.166	0.694	0.226	0.714
	Y	4.14	2.12	0.689	0.684	0.442	0.648	—	—	0.782	0.638
	M24	4.00	2.00	0.828	0.451	0.658	0.426	0.845	0.392	—	—
Within-crypt dist.	I	0.61	0.63	0.867	0.840	0.778	0.796	0.857	0.830	0.910	0.889
	D	1.22	1.46	0.304	0.042	0.203	0.030	0.294	0.035	0.408	0.053
	F	1.37	1.27	0.389	0.155	0.236	0.111	0.334	0.116	0.510	0.193
	H	1.16	1.43	0.662	0.058	0.527	0.044	0.615	0.045	0.755	0.078
	M8	0.81	0.85	0.919	0.612	0.838	0.522	0.899	0.522	0.949	0.650
	X	1.51	0.94	0.226	0.434	—	—	0.186	0.363	0.337	0.496
	Y	1.42	0.94	0.457	0.468	0.294	0.384	—	—	0.580	0.495
	M24	0.63	0.47	0.964	0.939	0.924	0.916	0.951	0.914	—	—
Unmeth. patterns	I	3.57	2.37	0.468	0.522	0.508	0.640	0.448	0.588	0.470	0.484
	D	3.29	2.36	0.402	0.331	0.437	0.439	0.373	0.388	0.414	0.282
	F	1.78	2.22	0.906	0.924	0.901	0.941	0.884	0.926	0.907	0.920
	H	4.83	3.19	0.064	0.400	0.071	0.500	0.049	0.434	0.053	0.368
	M8	0.78	2.33	0.964	0.864	0.957	0.892	0.946	0.862	0.955	0.846
	X	13.0	4.51	0.277	0.982	—	—	0.260	0.977	0.265	0.977
	Y	10.3	4.72	0.239	0.944	0.247	0.947	—	—	0.204	0.940
	M24	8.33	11.0	0.475	0.153	0.442	0.248	0.410	0.189	—	—
Singletons	I	0.71	0.76	0.866	0.881	0.713	0.810	0.884	0.890	0.886	0.886
	D	0.71	0.76	0.854	0.881	0.720	0.813	0.871	0.890	0.872	0.881
	F	1.56	1.51	0.484	0.339	0.223	0.188	0.548	0.367	0.520	0.323
	H	1.33	2.34	0.686	0.032	0.464	0.014	0.723	0.042	0.720	0.035
	M8	1.11	2.09	0.830	0.040	0.613	0.013	0.854	0.042	0.860	0.032
	X	4.00	2.65	0.003	0.024	—	—	0.024	0.052	0.004	0.021
	Y	0.86	1.46	0.975	0.518	0.825	0.261	—	—	0.977	0.488
	M24	1.50	1.87	0.678	0.170	0.287	0.051	0.832	0.243	—	—

[a] observed value. [b] value of the posterior probability distribution function computed on the data set indicated in subscripts $\mathbb{P}(X_{obs} \leq X_{pred})$. [c] each set correspond to a patient excepted for patient M that have both a 8 cells and a 24-cell dataset. [d] and [e] report the between crypts average and standard deviation summary statistics.

Table S1.1: Summary statistics computed separately for each patient when excluding data from either patient X, Y or M. Values of the probability distribution function that fall outside of the 95% confidence interval are underlined.

	MXY[a]				MY				MX				XY			
set[b]	ave[c]		sd[d]		ave		sd		ave		sd		ave		sd	
	obs.[e]	p[f]	obs.	p	obs.	p	obs.	p	obs.	p	obs.	p	obs.	p	obs.	p
Distinct patterns																
8	2.46	0.958	1.39	0.683	2.46	0.775	1.39	0.424	2.46	0.962	1.39	0.713	2.46	0.971	1.39	0.668
24	5.35	0.378	2.58	0.363	4.46	0.438	2.02	0.538	5.77	0.297	2.98	0.181	5.79	0.254	2.52	0.403
Polym. sites																
8	2.49	0.883	2.39	0.221	2.49	0.650	2.39	0.115	2.49	0.855	2.39	0.141	2.49	0.950	2.39	0.299
24	4.55	0.529	2.06	0.796	4.08	0.543	1.98	0.705	4.77	0.423	2.09	0.614	4.79	0.508	2.12	0.765
Average distance																
8	0.99	0.846	1.11	0.160	0.99	0.618	1.11	0.087	0.99	0.786	1.11	0.093	0.99	0.933	1.11	0.246
24	1.22	0.626	0.88	0.583	1.06	0.669	0.84	0.525	1.11	0.616	0.86	0.431	1.47	0.444	0.90	0.617
Unmeth. patterns																
8	2.70	0.731	2.74	0.718	2.70	0.736	2.74	0.859	2.70	0.650	2.74	0.774	2.70	0.705	2.74	0.665
24	10.6	0.238	7.02	0.972	9.38	0.299	7.93	0.836	10.8	0.282	8.17	0.857	11.6	0.166	4.65	0.998
Singletons																
8	0.97	0.975	1.36	0.513	0.97	0.786	1.36	0.218	0.97	0.983	1.36	0.562	0.97	0.981	1.36	0.510
24	2.15	0.309	2.41	0.011	1.15	0.622	1.62	0.098	2.85	0.165	2.58	0.024	2.43	0.194	2.62	0.006

[a]: indicates which of the 24-cell data are included in the analysis. [b]: labels 8 and 24 stand for the 8- and 24-cell datasets, respectively. [c,d]: results for both the empirical average and standard deviation of each summary statistic are reported here. [e,f]: observed values and the corresponding values of the posterior probability distribution functions $\mathbb{P}(X_{obs} \leq X_{pred})$.

Table S1.2: Values of the posterior probability distribution function for the five summary statistics evaluated when excluding the 24-cell data from either patient X, Y or M. The first four columns report the results obtained on the whole dataset. Values of the probability distribution function that fall outside of the 95% confidence interval are underlined.

S1.2 Impact of patient exclusion on the posterior of N

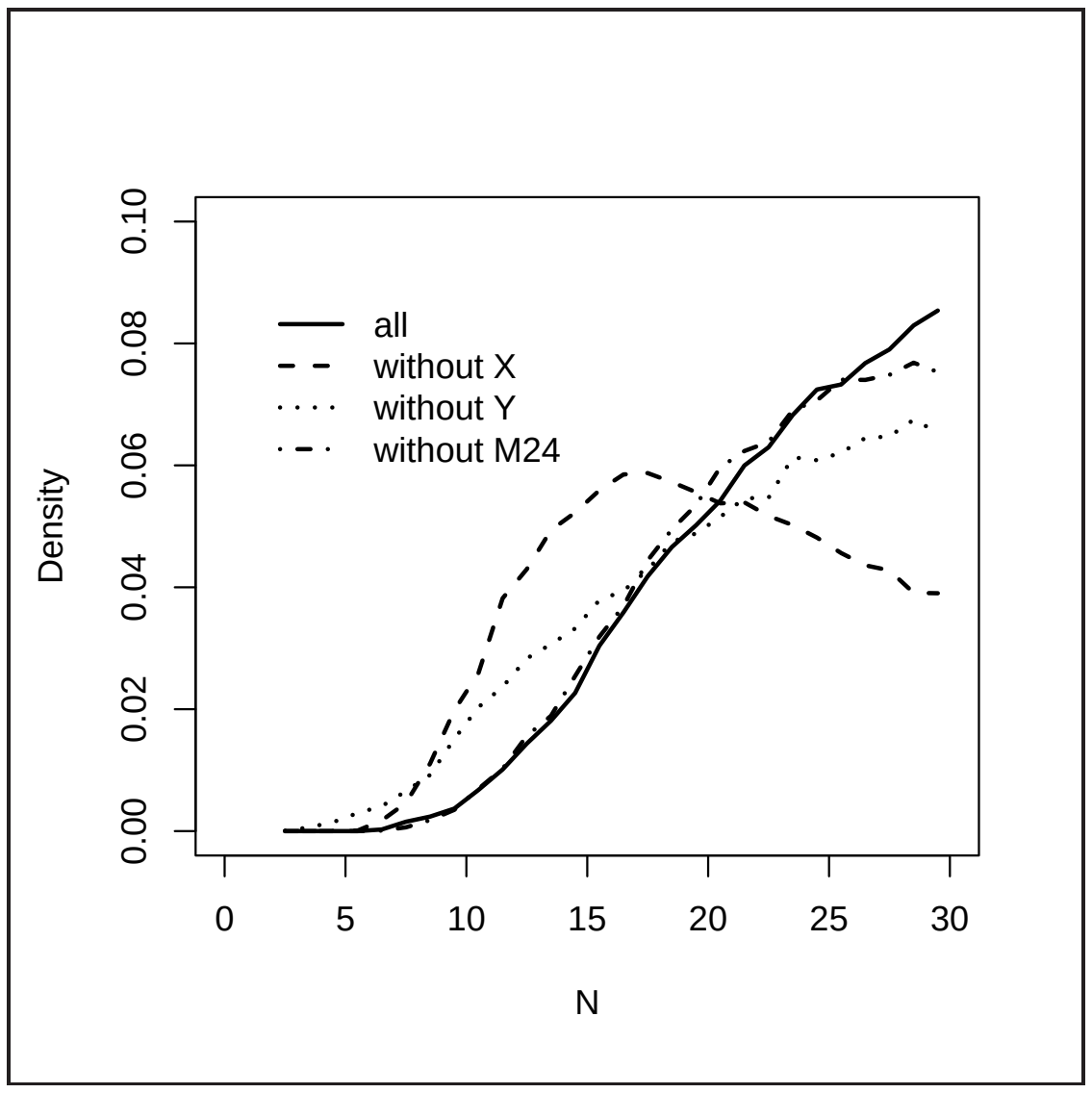

Figure S1.1: Posterior of N obtained after exclusion of either patient X, Y or M as compared to the posterior from the whole dataset.

The primary focus of this study is the posterior of N. We note in the main body of the manuscript that the exclusion of patient X makes the data compatible with a smaller value of N. We also examined the impact of the exclusion of Y or M on this posterior and the results are shown in Figure S1.1. Like the posterior obtained on the full data, these three posteriors only indicate lower bounds for N and ignoring data was found to result either in a shift towards lower values of N (patient X and Y) or to have no impact (patient M). The exclusion of X has the most impact and can therefore be seen as a conservative choice as it increases the range of N compatible with the data.

S1.3 Differences in the rate of the methylation process could explain the data from patient X

The question of the possible underlying biology that could explain the distinctive characteristics of the methylation patterns sampled from patient X is highly challenging given the amount of data available. In an attempt to address this question, we checked whether the distinctive characteristics could originate from rates of methylation/demethylation (parameter ν) specific to patient X.

Parameter ν was therefore estimated on data from patient X while all other parameters are kept fixed to values typical of their posterior on all data but those from patient X ($N = 15$, $\tau = 25$, $g = 7.5$, $\alpha = 0.01$ and $\epsilon = 0.001$). In a similar way, ν was also estimated on each other patient separately. Figure S1.2 show these posteriors and methylation/demethylation rates estimated on X actually happen to be much higher than those estimated on the other patients. We show in Table S1.3 than if we accept to take a specific set of rates for patient X (corresponding to the median of the posterior) then the model with $N = 15$, $\tau = 25$, $g = 7.5$, $\alpha = 0.01$ and $\epsilon = 0.001$ is compatible with the data from patient X. Of course, other explanations for the distinctive characteristics of the methylation patterns from patient X cannot be excluded.

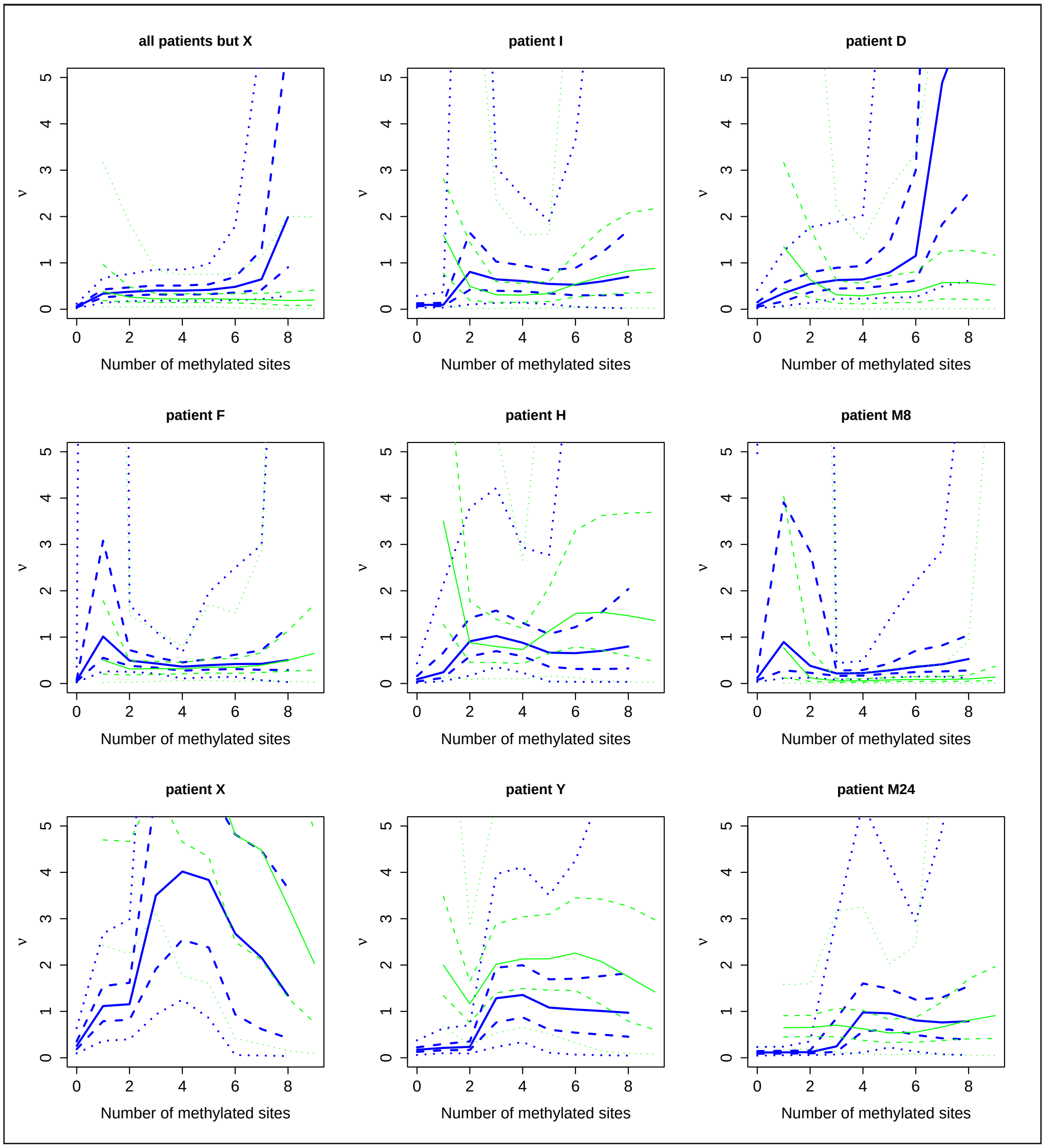

Figure S1.2: Posterior of ν obtained separately for each patient. The values of all parameters but ν are fixed: $N = 15$, $\tau = 25$, $g = 7.5$, $\alpha = 0.01$, $\epsilon = 0.001$.

Statistic	set	obs ave.	obs sd	X ave.	X sd
Distinct patterns	X	7.00	2.83	0.385	0.618
Polym. sites	X	5.43	2.07	0.500	0.708
Within-crypt dist.	X	1.51	0.94	0.406	0.474
Unmeth. patterns	X	13.0	4.51	0.501	0.841
Singletons	X	4.00	2.65	0.190	0.314

Table S1.3: Summary statistics for patient X as compared to their expected posterior for $N = 15$, $\tau = 25$, $g = 7.5$, $\alpha = 0.01$, $\epsilon = 0.001$ *and a specific set of methylation/demethylation rates. Specific rates were estimated as:* $\nu_m = (0.25, 1.10, 1.14, 3.59, 4.07, 3.88, 2.73, 2.21, 1.39, -)$ *and* $\nu_u = (-, 6.40, 6.33, 7.76, 6.81, 6.56, 4.89, 4.54, 3.27, 2.03)$.
