# Supplementary material for: The Stem Cell Population of the Human Colon Crypt: Analysis via Methylation Patterns
Source: PLoS Comput Biol. 2007 Mar 2;3(3):e28. doi: 10.1371/journal.pcbi.0030028 (PMC1808490; doi:10.1371/journal.pcbi.0030028)
Supplement: Supplementary file 2 [file pcbi.0030028.sd002.pdf]

Protocol S2. Supporting Materials and Methods

S2.1 Calculations in the context dependent model

Sequence evolution is usually modeled as a homogeneous continuous-time Markov chain along the genealogy, with independent sites. Transition probabilities are then computed for one site and the Markov chain that needs to be considered has a state space of the same size as the sequence alphabet (here 2). In more detail, the transition matrix P_t associated with an amount of time t is computed as $P_t = e^{Qt}$ where Q is the rate matrix. Exponentiation of the matrix Qt relies on the eigenvalue decomposition $Q = P^{-1}DP$ where D is diagonal. As long as the dimension n of the rate matrix (*i.e.* the dimension of the state space of the Markov chain) is small, both the eigenvalue decomposition (time-complexity $O(n^3)$, to be performed only once for a given Q) and the computation of P_t are fast (time-complexity $O(n^3)$ or $O(n)$ for a particular term of the matrix, to be performed for each t considered).

To account for context dependent effects using an homogeneous continuous-time Markov chain model, it is necessary to consider the state space of the $|\mathcal{A}|^l$ possible sequences (512 as in our case $|\mathcal{A}| = 2$ and $l = 9$). In the most general case, working with such a model is intractable given that each $O(n)$ or $O(n^3)$ computation needs to be performed a very large number of time during the course of the algorithm. However, when all sites are considered identical the time-complexity of the computations can be decreased considerably by taking advantage of some symmetries in the evolution models. For instance, the transition probability from a sequence x with k methylated sites to a sequence y depends on y only through $m(x, y)$, the number of sites methylated in y but unmethylated in x ($0 \leq m(x, y) \leq k$) and $u(x, y)$ the number of sites unmethylated in y but methylated in x ($0 \leq u(x, y) \leq l - k$). The same is true when looking at the transitions probability from any sequence to x. In other words, we can obtain the transition probabilities by working with the simplified Markov chain that describes transitions between sequences with different values of u and m in a state space of dimension $(k + 1)(l - k + 1)$ (at most 30 in our case).

The rate matrix Q_k of the simplified Markov chain is easily derived from the rates of methylation $(\mu_{+,0}, \ldots, \mu_{+,8})$ and demethylation $(\mu_{-,1}, \ldots, \mu_{-,9})$ for increasing numbers of methylated sites:

$$Q_k((m, u), (m', u')) = \begin{cases} u\mu_{+,k-u+m} & \text{if } m' = m \text{ and } u' = u - 1 \\ (k - u)\mu_{-,k-u+m} & \text{if } m' = m \text{ and } u' = u + 1 \\ m\mu_{-,k-u+m} & \text{if } m' = m - 1 \text{ and } u' = u \\ (l - k - m)\mu_{+,k-u+m} & \text{if } m' = m + 1 \text{ and } u' = u \\ 0 & \text{otherwise .} \end{cases}$$

S2.2 Genealogy of cells sampled from the progeny of the same stem cell.

We suppose that the progeny of each stem cell in the crypt, at any given time, is made of all the cells arising from the last g rounds of cell division. Here we seek to derive a continuous approximation for the discrete coalescent process corresponding to this scenario, illustrated in Figure 2.

The probability that two lineages sampled without replacement from the progeny of the same stem cell coalesce more than k generations ago is

$$P(T_2^{(2)} > k) = P(T_2 > k - 1) \times \frac{2^{g-k+1} - 2}{2^{g-k+1} - 1},$$

so that

$$P(T_2^{(2)} > k) = \frac{(2^g - 2^k)}{(2^g - 1)} \ , \quad 0 \le k \le g \ ,$$

where $T_2^{(2)}$ is the waiting time for the coalescent event. As mentioned in the Methods section, we prefer to rescale time in units of g generations and therefore we work with the random variable $S_2^{(2)} = T_2^{(2)}/g$ whose probability distribution function is defined by

$$P(S_2^{(2)} > q) \quad = \quad \frac{(2^g - 2^{gq})}{(2^g - 1)} \ , \quad q \in (0, 1/g, 2/g, \dots, 1) \ ,$$

We approximate the distribution of $S_2^{(2)}$ as a random variable taking values in the continuous interval $(0, 1)$ with probability distribution written $P(S_2^{(2)} > s) = \exp(-\Omega(s))$, where $\Omega(s) = \int_0^s \omega(a)da$ is the integrated rate function defined as

$$\Omega(s) \quad = \quad -\log\big(\frac{2^g - 2^{gs}}{2^g - 1}\big) \ , \quad 0 \le s < 1 \ .$$

We obtain ω after differentiating Ω

$$\omega(s) \quad = \quad g \log 2 \times \frac{2^{gs}}{2^g - 2^{gs}} \ , \quad 0 \le s < 1 \ .$$

The distribution of the coalescent time between two lineages sampled at time 0 follows the standard coalescent with constant rate 1 when expressed in terms of $u = \Omega(s)$. In this time scale, the distribution of the coalescent times has a well known generalization when n lineages are initially sampled[1] given by

$$f_n(u_n^{(n)}, \dots, u_2^{(n)}) = \prod_{j=2}^{n} \binom{j}{2} \exp\big(-\binom{j}{2}(u_j^{(n)} - u_{j+1}^{(n)})\big) \ , \quad 0 \le u_n^{(n)}, \dots, u_2^{(n)} < +\infty$$

where $u_j^{(n)}$ stands for the time spent with at least j lineages and with the convention $u_{n+1}^{(n)} = 0$. After changing back to the required time-scale, this density may be written:

$$f_n(s_n^{(n)}, \dots, s_2^{(n)}) = \prod_{j=2}^{n} \binom{j}{2} \omega(s_j^{(n)}) \exp\big(-\binom{j}{2}(\Omega(s_j^{(n)}) - \Omega(s_{j+1}^{(n)}))\big) \ , \quad 0 \le s_n^{(n)}, \dots, s_2^{(n)} < 1$$

where $s_j^{(n)}$ stands for the time spent with at least j lineages and with the convention $s_{n+1}^{(n)} = 0$. This choice ensures that for any $n \ge 2$ the time to the coalescent event for any pair of initially sampled lineages has the same distribution as $S_2^{(2)}$.

S2.3 A slightly modified model for the number of stem cells

MCMC moves trying to update the number of stem cells N without updating $\boldsymbol{M}$ have very low acceptation probability. This is understandable as M_i, the number of stem cells sampled in crypt i, has to verify $M_i \le N$ which implies that when $M_i = N$ for some i (almost always true if N is small and both the number of crypts K and the number of cell sampled are large) then moving from N to $N - 1$ is impossible.

This problem can be overcome by working with a slightly modified model where each crypt i has its own number of stem cells N_i ($\boldsymbol{N} \in \{(n_1, n_2, \dots, n_K), n_i \in (1, N_{max})\}$). The idea is that the posterior distribution of the parameters in the original model can be deduced from the posterior of the parameters in the modified model by conditioning on $\boldsymbol{N} \in \{(n, n, \dots, n), n \in$

[1] Kingman JFC (1982) On the genealogy of large populations. J. Appl. Prob. 19A:27–43.

$(1, N_{max})\}$. The size of the sample from the original posterior distribution that can be obtained through filtering of a sample from the modified posterior distribution is proportional to the posterior probability of $\{\boldsymbol{N} \in \{(n, n, \ldots, n), n \in (1, N_{max})\}\}$ in the modified model and then can be very small. We found that the prior on $\boldsymbol{N}$ such as

$$\mathbb{P}(\boldsymbol{N}) \propto \sum_{n=1}^{N_{max}} \frac{1}{\binom{K}{k}} \mathbb{I}\{|\boldsymbol{N} = n| = k, |\boldsymbol{N} = n+1| = K - k\} ,$$

where $|\boldsymbol{N} = n|$ denotes the number of elements of $\boldsymbol{N}$ that are equal to n, is a good choice as it ensures that $1/K$ of the prior weight is on the parameter space of the original model.

In practice we do not notice any significant differences between inferences in the original with uniform prior on $(1, N_{max})$ and inferences in the modified model using this prior on $\boldsymbol{N}$. As a consequence, the posterior distributions presented here are those obtained for the modified model and we identify N and $(1/K) \sum_{k=1}^{K} N_i$ when it is convenient.

S2.4 MCMC Algorithm

The MCMC algorithm generates a Markovian sample from the joint posterior distribution

$$\boldsymbol{N}, \tau, g, \sigma, \nu, \alpha, \epsilon, \boldsymbol{\Lambda}, \boldsymbol{Y} \mid \boldsymbol{X} .$$

Let us recall that $\boldsymbol{N} = (N_1, \ldots, N_K)$ denotes the number of stem cells in each crypts (N has been replaced by $\boldsymbol{N}$ to improve the algorithm performance, see above); τ is approximately the average time to the most recent common ancestor of the stem cell population; ν denotes the sequence evolution rates expressed in terms of number events in time τ; σ is the hyper-parameter; ϵ is the probability of error per sequence per site; $\boldsymbol{\Lambda}$ is the genealogy of the sampled cells; $\boldsymbol{Y}$ are the methylation patterns at the nodes and leafs of $\boldsymbol{\Lambda}$; $\boldsymbol{X}$ is the observed sequences that can differ from the patterns at the leafs of $\boldsymbol{Y}$ due to sequencing errors.

The MCMC algorithm is made of numerous small moves updating the variables while preserving the target posterior distribution. Most of these moves are Metropolis-Hastings steps where a new value for the variable is proposed according to a proposal distribution and the new value is accepted according to a probability that ensures the preservation of the target distribution. Some of the moves are Gibbs steps where one variable is updated by redrawing it from its distribution conditionally on all other variables.

Updating $\boldsymbol{N}, \tau, g, \sigma, \nu, \alpha, \epsilon$

Two kinds of Metropolis-Hastings moves have been designed to update $\boldsymbol{N}$. The first move consists of increasing or decreasing N_k by one in the crypt k. The second move increases or decreases N_k simultaneously in all the crypts $1 \leq k \leq K$. The parameters $\tau, g, \sigma, \nu, \alpha$ are updated separately by Metropolis-Hastings moves using a Gaussian proposal with standard deviation 0.2 times the current value of the parameter. The parameter ϵ is updated using a Gibbs move as its conditional distribution is known: $\epsilon \mid \ldots \sim Beta(1 + |\boldsymbol{X} \neq \boldsymbol{Y}|, 1 + |\boldsymbol{X} = \boldsymbol{Y}|)$, where $|\boldsymbol{X} \neq \boldsymbol{Y}|$ denotes the number of CpG sites where $\boldsymbol{X}$ and $\boldsymbol{Y}$ does not match each other.

In the case of the context-dependent sequence evolution model we have an additional set of parameters corresponding to the boundaries of the range where each couple of methylation/demethylation rate apply. To update these boundaries we choose one of them at random and move it to the left or to the right. The Metropolis-Hastings ratio for this move is easy to compute.

Updating $\boldsymbol{\Lambda}$, $\boldsymbol{Y}$ and $\boldsymbol{N}$

These moves are attempted separately and independently for each crypt. We will describe them as if we were analyzing a single crypt to avoid useless notation.

Updating $\boldsymbol{Y}$. Move (a) updates Y_i, in turn for each node i of the genealogy Λ by redrawing it from its distribution conditionally on all other variables (Gibbs move):

$$\pi(Y_i = y \mid \ldots) \propto f(y; y_p, t_p) f(y_l; y, t_l) f(y_r; y, t_r) \ ,$$

where the subscript p corresponds to the parent of node j; the subscript l to its left child; the subscript r to its right child; and $f(y; x, t)$ to probability of the transition from x to y along a branch of length t.

In a similar way, patterns at the leafs of Λ are updated using the formula:

$$\pi(Y_i = y \mid \ldots) \propto f(y; y_p, t_p) h(x; y) \ ,$$

where $h(x; y)$ is the probability of observing the sequence x when the true underlying sequence is y ($h(x; y) = (l - e)^{1-\epsilon} e^{\epsilon}$ where e is the number of sequencing errors and l the number of CpG sites).

Updating $\boldsymbol{\Lambda}, \boldsymbol{Y}, \boldsymbol{N}$.

The genealogy and patterns at its node are jointly updated using the branch-swapping strategy proposed by Wilson and Balding[2] and used in the BATWING program. Briefly, it consists of choosing a node i and trying to move its parent node somewhere else in the genealogical tree. The powerful idea of Wilson and Balding is to choose a node j above which to try to attach the parent according to the distance between the sequences Y_i and Y_j, thereby making possible to achieve relatively large moves with a relatively high frequency. In keeping with the original algorithm, we choose j using a proposal density proportional to $1/(1 + d(Y_i, Y_j))$, where d denotes the Hamming distance. In our version of the branch-swapping move, we update the sequence of the parent node according to its conditional distribution in the new topology. N is redrawn from its conditional distribution given the new Λ and the number of stem cells in the other crypts. The Metropolis-Hastings acceptance ratio is easy to compute.

Another move updates the number of roots of Λ. With equal probabilities we try either to decrease or to increase the number of roots. To decrease the number of roots we randomly choose two of them to merge. The time of the new coalescent event added to the genealogy is chosen uniformly between the time of the last coalescent event and the time of the birth of the patient. The sequence at the new internal node is drawn according to its conditional distribution in the new genealogy. To increase the number of roots we try to replace the last internal node of the genealogy by two root nodes. The Metropolis-Hastings acceptance ratio is easy to compute.

S2.5 Parameters used to generate simulated data sets

N	τ	g	α	ϵ	$\nu_m(0)$	$\nu_m(1)$	$\nu_m(2)$	$\nu_m(3)$	$\nu_m(4)$	$\nu_m(5)$	$\nu_m(6)$	$\nu_m(7)$	$\nu_m(8)$	—
					—	$\nu_u(1)$	$\nu_u(2)$	$\nu_u(3)$	$\nu_u(4)$	$\nu_u(5)$	$\nu_u(6)$	$\nu_u(7)$	$\nu_u(8)$	$\nu_u(9)$
6	32.2	6.05	0.082	0.001	0.069	0.337	0.360	0.528	0.522	0.530	0.566	0.855	21.534	—
					—	0.949	0.785	0.306	0.285	0.286	0.276	0.257	0.251	0.287
24	31.6	7.35	0.018	0.002	0.066	0.369	0.386	0.511	0.509	0.524	0.589	0.875	6.222	—
					—	0.868	0.753	0.312	0.280	0.285	0.267	0.262	0.242	0.327

Table S2.5: Parameters used to generate simulated data sets.

[2] Wilson IJ, Balding DJ (1998) Genealogical inference from microsatellite data. Genetics 150:499-510.
