# Supplementary material for: The Stem Cell Population of the Human Colon Crypt: Analysis via Methylation Patterns
Source: PLoS Comput Biol. 2007 Mar 2;3(3):e28. doi: 10.1371/journal.pcbi.0030028 (PMC1808490; doi:10.1371/journal.pcbi.0030028)
Supplement: Supplementary file 3 [file pcbi.0030028.sg001.pdf]

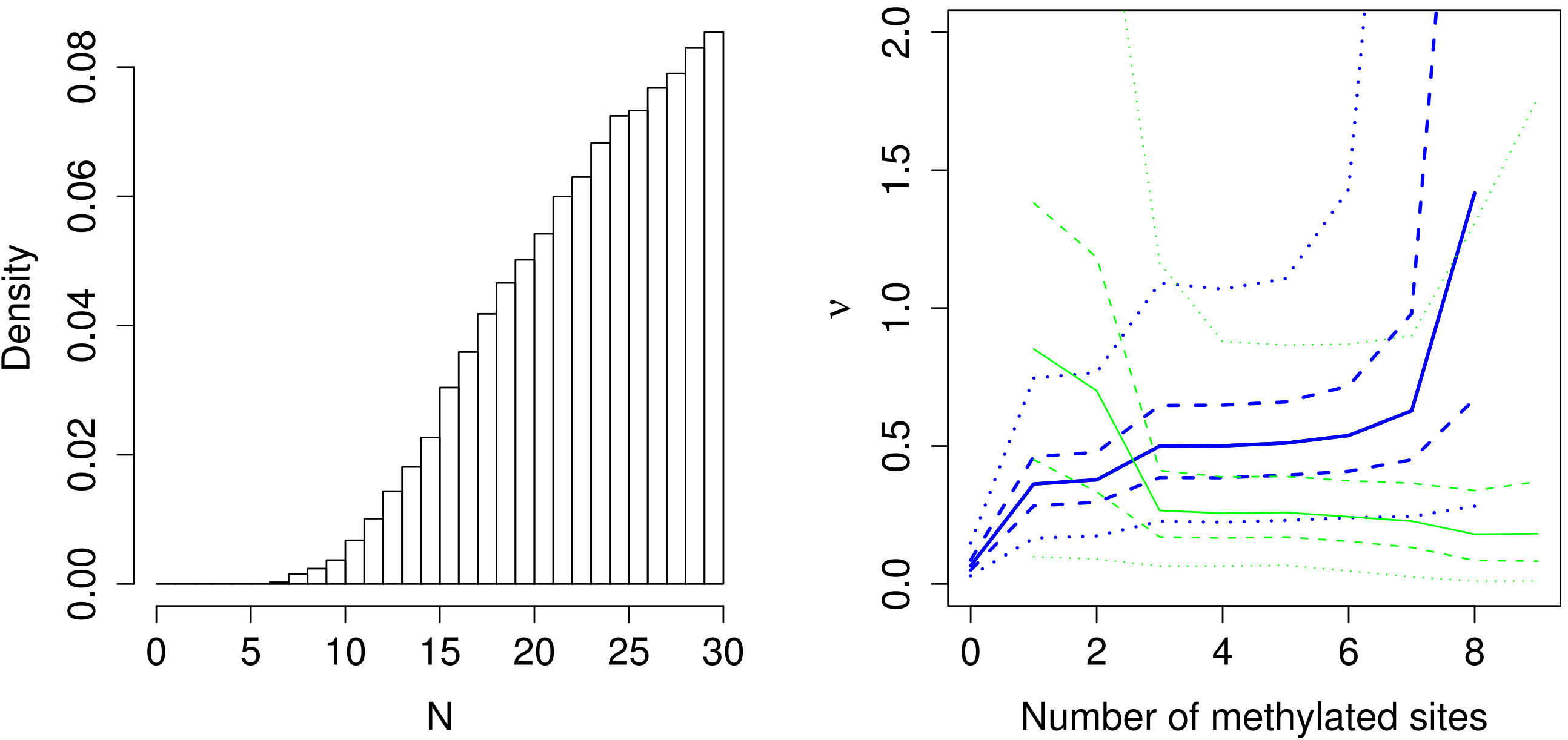

Density
0.00
0.02
0.04
0.06
0.08
0 5 10 15 20 25 30
N
ν
0.0
0.5
1.0
1.5
2.0
0 2 4 6 8
Number of methylated sites
