# Supplementary material for: The Stem Cell Population of the Human Colon Crypt: Analysis via Methylation Patterns
Source: PLoS Comput Biol. 2007 Mar 2;3(3):e28. doi: 10.1371/journal.pcbi.0030028 (PMC1808490; doi:10.1371/journal.pcbi.0030028)
Supplement: Supplementary file 4 [file pcbi.0030028.sg002.pdf]

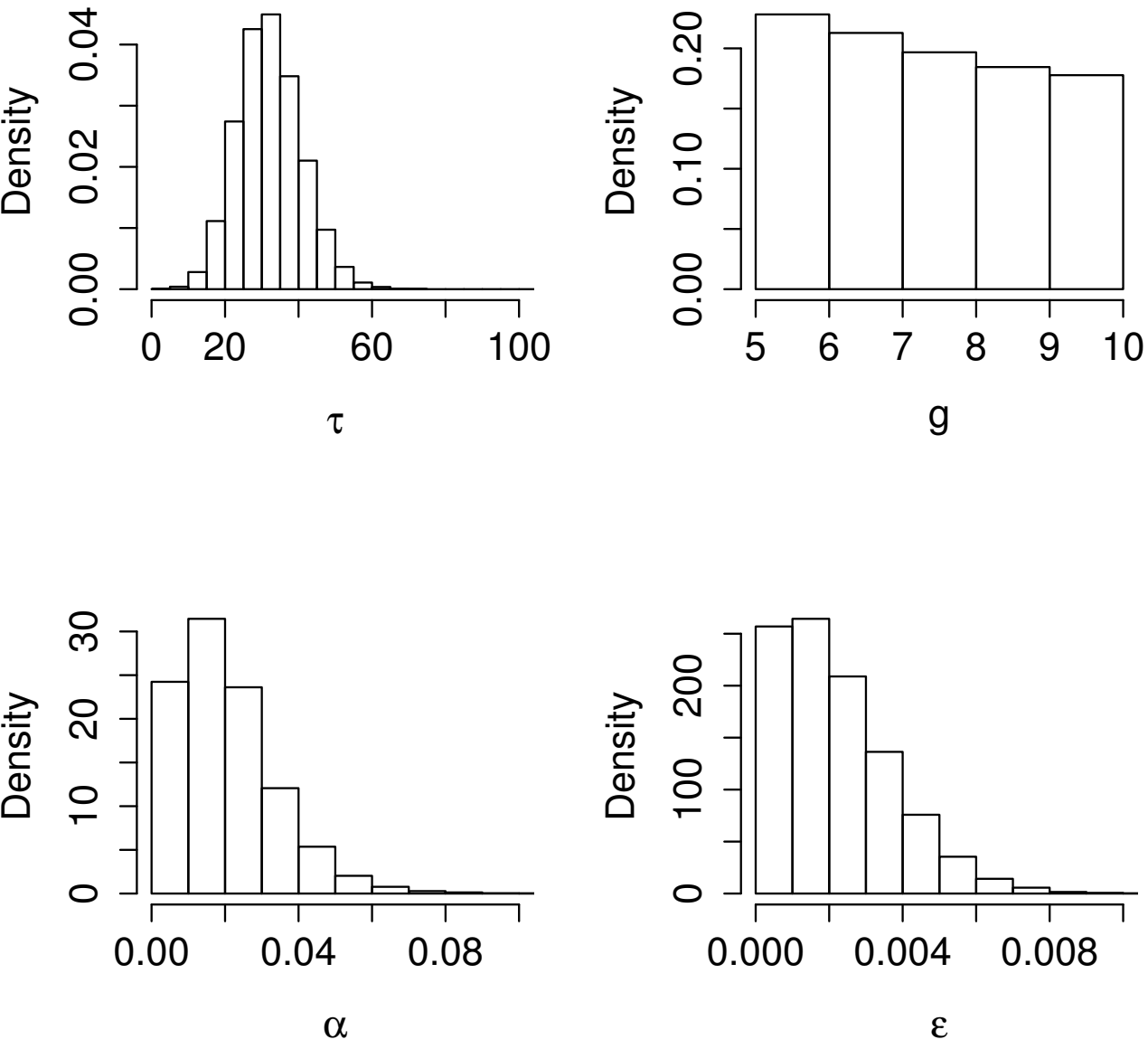
Density
0.00
0.02
0.04
0
20
60
100
τ
Density
0.00
0.10
0.20
5
6
7
8
9
10
g
Density
0
10
20
30
0.00
0.04
0.08
α
Density
0
100
200
0.000
0.004
0.008
ε
