# Supplementary material for: The Stem Cell Population of the Human Colon Crypt: Analysis via Methylation Patterns
Source: PLoS Comput Biol. 2007 Mar 2;3(3):e28. doi: 10.1371/journal.pcbi.0030028 (PMC1808490; doi:10.1371/journal.pcbi.0030028)
Supplement: Supplementary file 6 [file pcbi.0030028.sg004.pdf]

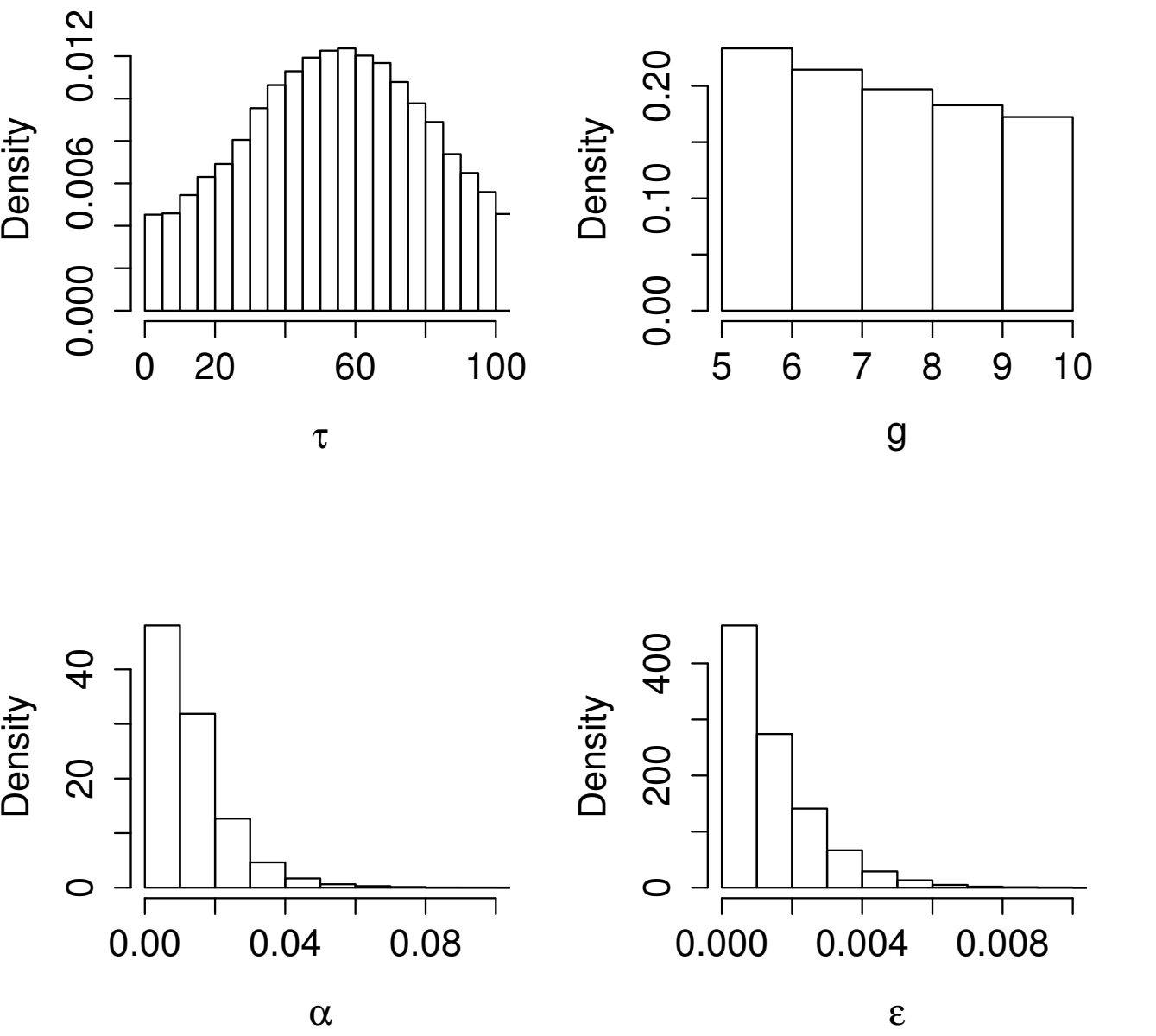
Density
0.000
0.006
0.012
0 20 60 100
τ
Density
0.00
0.10
0.20
5 6 7 8 9 10
g
Density
0
20
40
0.00 0.04 0.08
α
Density
0
200
400
0.000 0.004 0.008
ε
